# Overexpression of WWP1 Promotes tumorigenesis and predicts unfavorable prognosis in patients with hepatocellular carcinoma

**DOI:** 10.18632/oncotarget.5712

**Published:** 2015-10-17

**Authors:** Xiao-Fei Zhang, Jie Chao, Qiu-Zhong Pan, Ke Pan, De-Sheng Weng, Qi-Jing Wang, Jing-Jing Zhao, Jia He, Qing Liu, Shan-Shan Jiang, Chang-Long Chen, Hong-Xia Zhang, Jian-Chuan Xia

**Affiliations:** ^1^ State Key Laboratory of Oncology in Southern China, Collaborative Innovation Center for Cancer Medicine, Sun Yat-sen University Cancer Center, Guangzhou 510060, People's Republic of China; ^2^ Department of Biotherapy, Sun Yat-Sen University Cancer Center, Guangzhou 510060, People's Republic of China; ^3^ Shanxi Entry-Exit Inspection and Quarantine Bureau, Shanxi, 710068, People's Republic of China

**Keywords:** WWP1, hepatocellular carcinoma, prognosis, oncogene

## Abstract

WW domain-containing E3 ubiquitin protein ligase 1 (WWP1) has been speculated to play important roles in the development of several kinds of cancers. However, the role of WWP1 in hepatocellular carcinoma(HCC) is not clear. In the present study, we investigated the expression and prognostic role of WWP1 in primary hepatocellular carcinoma (HCC) using cell lines and 149 archived HCC samples. Correlation between the functions of WWP1 in HCC was also explored. We used human HCC cell lines (BEL-7402, SMMC-7721, Hep-G2, Hep-3B, SK-hep1 and Huh7) and a normal hepatocyte cell line (LO2) along with HCC samples from patients who had undergone resection for HCC previously at our hospital. A battery of methods (real-time quantitative polymerase chain reaction; western blotting; immunohistochemical analyses; cell proliferation and colony formation assays; cell migration and cell invasion assays) were employed to assess various aspects of WWP1. We found that WWP1 expression was upregulated aberrantly at mRNA and protein levels in human primary HCC tissues. Amplified expression of WWP1 was highly correlated with poor outcome. Silencing of WWP1 expression by siRNA inhibited the proliferation, colony formation, migration and invasion of HCC cells *in vitro*, and resulted in significant apoptosis and cycle arrest in HCC cells. Our findings suggest that WWP1 might have an oncogenic role in human primary HCC, and that it could be used as a prognostic marker as well as a potential molecular target for the treatment of HCC.

## INTRODUCTION

Hepatocellular carcinoma (HCC) constitutes a major global health problem. It is the dominant form of primary liver cancer and the third leading cause of cancer-related deaths worldwide, accounting for over half a million deaths annually [1]. The incidence of HCC varies significantly by geographic region: it is prevalent in Southeast Asia and sub-Saharan Africa where the hepatitis B virus (HBV) is endemic; and is the second most common malignancy in China, accounting for approximately 350,000 deaths per year [2, 3, 4, 5]. Despite recent developments in therapeutic strategies including surgical resection, radiotherapy and chemotherapy, the prognosis of patients with HCC remains poor due to the absence of early symptoms and rapid tumor progression and invasion during the early stages [6, 7, 8]. Tumor occurrence, development and metastatic potential are frequently linked to altered profiles in gene expression; therefore identifying potential biological markers for early diagnosis and novel therapeutic strategies is central to improving prognosis in patients with HCC [9].

WW domain-containing E3 ubiquitin protein ligase 1 (WWP1), also known as TGIF-interacting ubiquitin ligase 1 (TIUL1) [10] or Atropin-1-interacting protein 5 (AIP5) [11], is a neural precursor cells-expressed developmentally down-regulated 4-like E3 ubiquitin-protein ligase (NEDD4) [9]. Ubiquitination proceeds through a three-step cascade involving three classes of enzymes [12]: E1 ubiquitin-activating enzyme that activates ubiquitin via an ATP-dependent reaction; E2 ubiquitin-conjugating enzyme that transfers the activated ubiquitin moiety from E1 to an E3 ubiquitin ligase; and E3 enzymes that physically interact with substrates and therefore critical determinants in the specificity of ubiquitination [13]. WWP1 is an intrinsic E3 ubiquitin ligase contain an N-terminal C2 domain, four tandem WW domains and a C-terminal HECT (homologous to the E6-associated protein carboxyl terminus) domain. The N-terminal C2 domain is responsible for calcium-dependent phospholipid binding; the four middle WW domains recognize substrates with proline-rich (PY or PPXY) motifs; and the C-terminal HECT domain acts as the catalytic center for ubiquitin transfer [14, 15].

Human WWP1 is localized on chromosome 8q21, a region frequently amplified in many human cancers. The copy number gain of WWP1 has been reported in 51% of breast cancer cell lines; 41% of primary breast tumors; 35% of oral cancer samples; 44% of prostate cancer cell lines and xenografts; and 31% of clinical prostate cancer samples [9]. It has been implicated in the regulation of various regulatory and signaling processes involved in tumor proliferation and apoptosis. These include proteasome-dependent degradation of specific substrates and tumor suppressors, such as p53 by Mdm2 and Pirh2 ligases [16, 17, 18]; PTEN by NEDD4 [18, 19, 20]; and p73 and p63 by Itch [22, 23, 25]. WWP1 plays regulatory roles in receptor signaling, notably negative regulation of transforming growth factor-β (TGF-β) signaling through interaction with Smad7 leading to the ubiquitylation and degradation of TGF-β receptor type 1 [25, 26], degradation of Smad2 via TGF-β-induced factor (TGIF) [27], regulation of senescence, bone differentiation and metastasis via ring finger protein 11 (RNF11). More recently, WWP1 has been shown to promote the activities of ErbB2 and EGF receptors [28].

Previous investigations have shown that overexpression of WWP1 could promote cell growth, whereas depletion of WWP1 suppressed proliferation and induced apoptosis in breast cancer cells [29, 30], prostate cancer cells [32] and oral cancer cells [33]. The growth promoting activity of WWP1 has been demonstrated in MDCK canine kidney epithelial cells [38], PC-3 prostate cancer cells [32], SC3, CA922, CAL27, TW206, SAS and OECM-1 oral cancer cell [33] and HCT116 colon cancer cells [24]. In contrast, a deficiency of WWP1 in HCT116 cells was sensitized the cells to chemotherapeutic drugs, such as doxorubicin and cisplatin [24]. Despite these extensive investigations, the prognostic significance of WWP1 in human HCC remains unclear. Therefore, the present study was conducted to elucidate the molecular mechanisms underlying expression of WWP1 and its clinical significance in human HCC.

## RESULTS

### Expression of WWP1 in human HCC tissues

**Figure 1:**
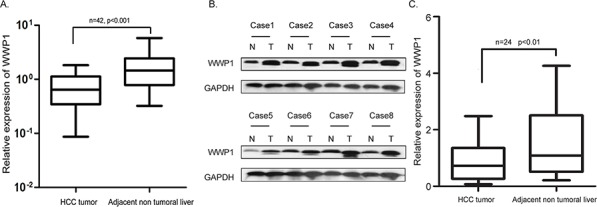
Expression of WWP1 mRNA and protein in human primary HCC surgical specimens as evaluated by real-time quantitative PCR and western blot **A.** Relative mRNA expression of WWP1 was higher in 42 HCC tissues than in matched adjacent non-cancerous tissues (*P* < 0.01). **B.** Representative result of expression of WWP1 protein in eight paired HCC tissues and matched adjacent non-cancerous tissues (T, HCC tissues; N, matched non-cancerous tissues). **C.** Relative expression of WWP1 protein was increased remarkably in 24 HCC tissues than in matched adjacent non-cancerous tissues (*P* < 0.01).

Quantitative RT-PCR showed that tissues from primary human HCC biopsies exhibited significantly higher expression rates of WWP1 mRNA compared to adjacent non-tumor tissues (30/42; 70.4%; *P* = 0.0003; Figure 1A). In general, higher levels of mRNA lead to increased expression of its encoded protein. In order to verify this link in the case of WWP1 expression, the HCC tissue samples were subjected to Western blot analysis. As shown in Figure 1B and 1C, expression of WWP1 protein was significantly elevated in 14/24 (58.3%) specimens of human primary HCC tissue compared to compared to adjacent non-tumor hepatic tissues (*P* = 0.004).

### Correlations between expression of WWP1 in tumor tissues and clinicopathologic features in patients with HCC

**Figure 2:**
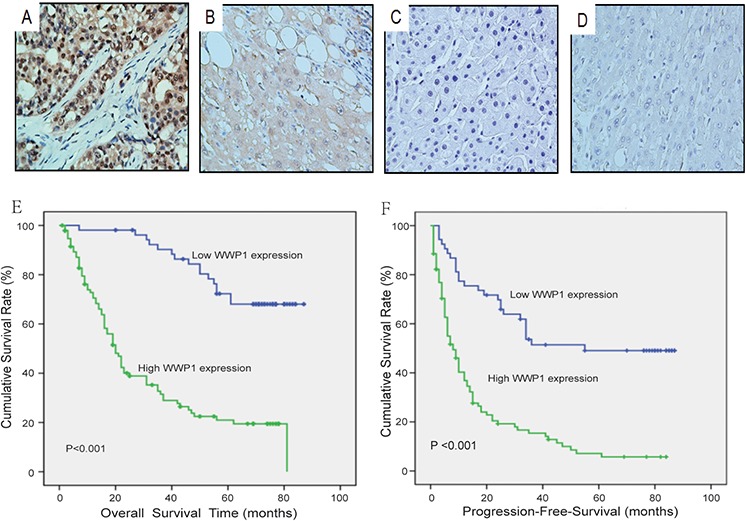
Immunohistochemical analyses of WWP1 protein expression in primary hepatocellular carcinoma surgical specimens and Kaplan–Meier survival analyses of the primary HCC patients (*n* = 149) with high WWP1 expression (*n* = 95) and low WWP1 expression (*n* = 54) after resection **A.** Strong WWP1 staining was observed in poorly differentiated HCC, scored as WWP1 (+++). **B.** Intermediate WWP1 staining in moderately differentiated HCC, scored as WWP1 (++). **C.** Weak WWP1 staining in poorly differentiated HCC, scored as WWP1 (+). **D.** WWP1-negative staining in normal liver tissue distant from the tumor, scored as WWP1 (−). **E–F.** Based on their WWP1 immunostaining scores, HCC patients were divided into low-WWP1 expression (WWP1− or WWP1+) and high-WWP1 expression (WWP1++ or WWP1+++) groups. Overall survival (E) and Progression- Free-Survival (F) of patients in the high-WWP1 group was significantly lower than that of patients in the low-WWP1 group (*P* < 0.001 for the log-rank test).

Immunohistochemical analysis confirmed that WWP1 expression was positive in primary HCC tissue samples (Figure 2A–2C) but was negative in adjacent non-tumor tissues (Figure 2D). Furthermore, expression of WWP1 increased in a stepwise fashion from well-differentiated to moderately-differentiated to poorly-differentiated HCC specimens. Kaplan-Meier survival analyses revealed that patients with high levels of WWP1 expression had significantly poorer overall survival (OS) and progression free survival (PFS) times than those with low expression levels (Figure 2E and 2F: *P* < 0.001). The correlations between WWP1 expression and various clinicopathologic parameters are summarized in Tables 1 and 2. Chi-square analysis revealed that the expression of WWP1 in HCC tissues was highly correlated with tumor size (*P* = 0.015), histological grade (*P* < 0.001), TNM stage (*P* < 0.001), vascular invasion (*P* = 0.018) and tumor capsule (*P* = 0.026) but not with age, sex, HBV, serum AFP or liver cirrhosis (Table 1). Univariate Cox regression analysis showed that WWP1 expression and TNM stage were significantly associated with OS in patients with HCC; and multivariate Cox regression analysis indicated that WWP1 expression was an independent predicator of OS in HCC (*P* < 0.001; Table 2).

**Table 1:** Correlation between WWP1 expression and clinicopathological variables of 149 patients with hepatocellular carcinoma

Clinicopathologic variable	Number in each group	WWP1 expression		*P*
		Low	High	
All cases	149	54	95	
Age (years)				0.879
<50	84	30	54	
≥50	65	24	41	
Sex				
Male	122	44	78	0.924
Female	27	10	17	
Tumor size (cm)				0.015^a^
<5	66	31	35	
≥5	83	23	60	
Histological grade				<0.001^a^
Well	26	19	7	
Moderate	94	28	66	
Poor	29	7	22	
Liver cirrhosis				0.668
No	25	10	15	
Yes	124	44	80	
HBV				0.660
Negative	16	5	11	
Positive	133	49	84	
Serum AFP (μg/L)				0.223
<25	82	42	40	
≥25	67	41	26	
TNM stage				<0.001^a^
I	91	43	48	
II–III	58	11	47	
Vascular invasion				0.018^a^
No	131	52	79	
Yes	18	2	16	
Tumor capsule				0.026^a^
No	76	21	55	
Yes	73	33	40	

AFP, alfa fetoprotein; HBV, hepatitis B virus.

a *P* < 0.05.

**Table 2:** Univariate and multivariate analyses of overall survival in hepatocellular carcinoma

Variable	Univariate analyses			Multivariate analyses		
	HR	95% CI	*P*	HR	95% CI	*P*
WWP1	5.322	3.096–9.149	<0.001^a^	4.992	2.882–8.647	<0.001^a^
Age	0.907	0.735–1.120	0.364			
Sex	1.019	0.776–1.336	0.894			
Tumor size	0.853	0.689–1.056	0.144			
Histological grade	0.895	0.437–1.183	0.437			
Liver cirrhosis	0.474	0.838–1.463	0.474			
HBV	1.082	0.766–1.528	0.656			
Serum AFP	1.070	0.864–1.326	0.534			
TNM stage	1.870	1.220–2.865	0.004^a^	1.845	1.680–2.049	0.045^a^

HR, hazard ratio; CI, confidence interval; AFP, alfa fetoprotein; TNM, tumor, node, metastasis

a *P* < 0.05.

### Correlations between WWP1 expression and prognosis in patients with primary HCC

To determine the prognostic value of WWP1 in patients who had undergone surgery for primary HCC, both OS and PFS were evaluated using the Kaplan-Meier method. The results showed that mean OS and PFS in patients with low levels of WWP1 expression were 71 and 34 months, respectively, compared to 19 and 7 months in patients with high levels of WWP1 expression. These results indicated that patients with elevated levels of WWP1 had significantly shorter OS times (*P* = 0.0005; Figure 2E) and a greater propensity for disease recurrence (*P* = 0.0034; Figure 2F) than those with low levels. By the time of the final follow-up, a total of 87/149 (58.4%) patients had died.

**Figure 3:**
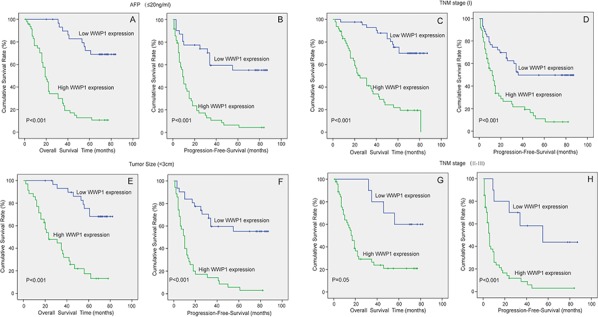
Correlation of WWP1 expression with OS and PFS in HCC subgroups Analyses of OS and PFS by AFP ≤ 25 ng/ml **A, B.** TNM stage **C, D.** and **G,H.** and Tumor Size < 3 cm **E, F.**

These observations were further confirmed by stratified OS and PFS analyses: OS and PFS in patients with high expression of WWP1 were found to be dependent on AFP concentration (Figure 3A and 3B), TNM stage (Figure 3C–3D and 3G–3H) and tumor size (Figure 3E and 3F). The threshold levels were set at AFP ≤ 25 ng/ml, TNM stage: I *vs*. stage II–II and tumor size < 3 cm.

### Inhibition of WWP1 expression in HCC cell lines

**Figure 4:**
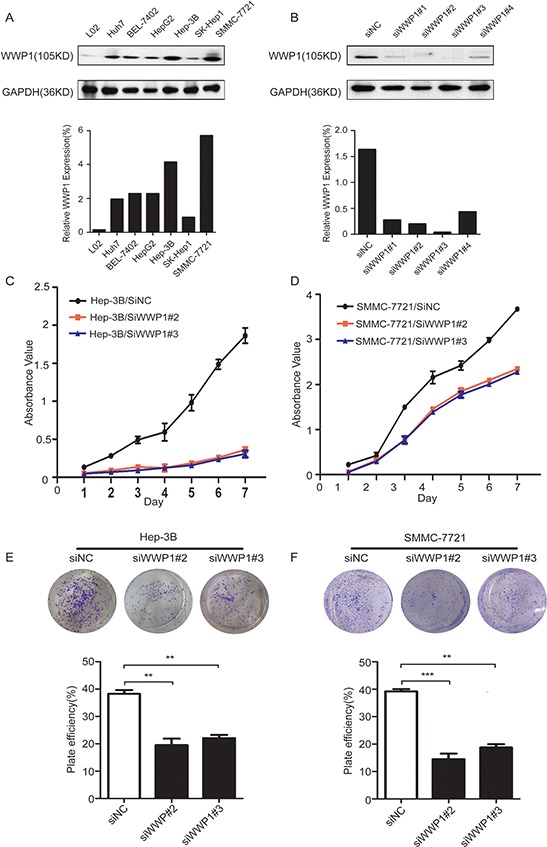
Expression of WWP1 protein in human HCC cell lines and the efficiency of WWP1-targeting siRNAs in Hep-3B and SMMC-7721 cell lines And growth-promoter role of WWP1 in cell proliferation and colony-formation assays of Hep-3B and SMMC-7721 cell lines. **A.** WWP1 protein was upregulated in Huh7, BEL-7402, HepG2, Hep3B, SK-hep1 and SMMC-7721 cells (particularly in Hep-3B and SMMC-7721 cells) compared with the normal liver cell line LO2. **B.** siWWP12# and siWWP1#3 had the highest knockdown efficiency of the four tested siRNAs. **C, D.** Cell-proliferation assay showing the suppressive effect of knocking down WWP1 expression in *vitro* proliferation of Hep-3B (A) and SMMC-7721 (B) cell lines. Experiments were done in triplicate. E and F. Representative inhibition of colony formation in a monolayer culture by inhibition of WWP1. Bottom, quantitative analyses of foci numbers are shown as the mean ± standard deviation. Experiments were carried out in triplicate. *P*-values were calculated using the Student's *t*-test. **P < 0.01 *versus* control; ***P < 0.001 *versus* control.

Western blot analysis revealed that WWP1 protein expression was upregulated in six tested HCC cell lines (BEL-7402, SMMC-7721, Hep-G2, Hep-3B, SK-hep1 and Huh7) compared to the corresponding level in normal hepatic cell line LO2. The relative expression levels were highest in SMMC-7721 and Hep-3B cell lines (Figure 4A). The effects of knocking down WWP1 on WWP1 protein expression was shown in Figure 4B. This was demonstrated by employing four WWP1-targeting siRNAs (siWWP1#1, siWWP1#2, siWWP1#3 and siWWP1#4). Quantification revealed that siWWP1#2 and siWWP1#3 were more effective in silencing WWP1 than siWWP1#1 and siWWP1#4 (Figure 4B).

### Knockdown of WWP1 inhibited cell growth in HCC *in vitro*

To explore the metabolic actions of WWP1 in HCC cells, a series of experiments were performed after silencing WWP1 with siRNAs to examine the potential consequences of loss-of-function in the tumorigenic phenotypes. Both cell proliferation and colony-forming assays demonstrated that the growth capacity of HCC cells was significantly reduced 48 h post-transfection compared to that in negative control (siNC) cells (Figure 4C–4D and 4E–4F, respectively).

### WWP1 silencing suppressed migration and invasion in HCC cells *in vitro*

**Figure 5:**
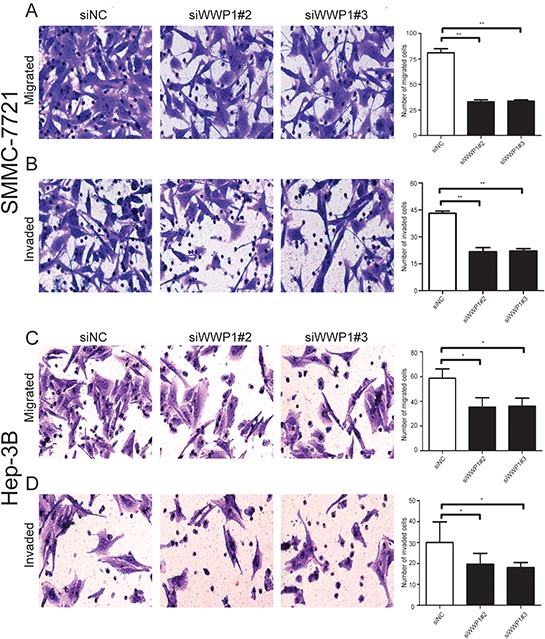
Transwell migration assays and Matrigel invasion assays of SMMC-7721 and Hep-3B cells transfected with WWP1-specific siRNAs and control siRNA Images are shown on the left (×100 magnification) and quantification of ten randomly selected fields on the right. Data are the mean ± SD of three independent experiments. *P*-values were calculated using the Student's *t*-test. **A, C.** WWP1 knockdown inhibited migration of SMMC-7721 (A) and Hep-3B (C) cells. **B, D.** WWP1 inhibition significantly attenuated invasion of SMMC-7721 (B) and Hep-3B (D) cells. *P < 0.05 versus siControl; **P < 0.01 versus siControl.

Correlation analyses had revealed that overexpression of WWP1 in HCC tissue specimens was significantly associated with clinical stage (*P* < 0.001). Subsequent analyses to determine the effect on metastasis in HCC was carried out through migration and Matrigel invasion assays. Both assays demonstrated that the migration and the invasive capability of HCC cells (Hep-3B and SMMC-7721) were significantly reduced following WWP1 knockdown *in vitro* (Figure 5). These results indicated that WWP1 may promote cell invasion and metastasis in patients with HCC.

### WWP1 depletion suppressed cell cycle progression in HCC cell lines *in vitro*

**Figure 6:**
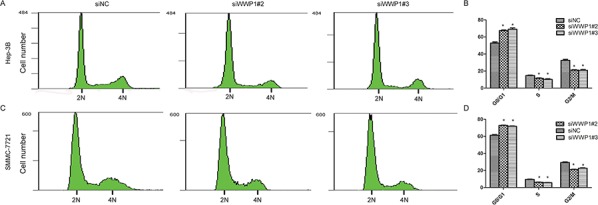
Depletion of WWP1 induces G1/S arrest of HCC cells Flow cytometry analysis of the indicated Hep-3B **A, B.** cells and SMMC-7721 **C, D.** transfected WWP1-specific siRNA(s). The proportion of S-phase cells as significantly reduced in WWP2 sIRNA(s)-transfected cell lines (*P* < 0.05) compared with the control group; in contrast, the proportion of S-phase and G2/M cells in the line transfected with the WWP1 construct clearly increased (C,D). (*P* < 0.05) *P < 0.05

To elucidate the potential mechanisms underlying the pro-proliferative effect of WWP1 in HCC cells, flow cytometric analysis of cell cycle distribution was performed in Hep-3B and SMMC-7721 cell lines following 72 h transfection with siWWP1s. The results revealed that knockdown of WWP1 led to cell cycle arrest at G0/G1 phase and reduced the percentages of cells at S and G2/M phase in both cell lines compared to the corresponding percentages in negative (siNC) control cells (Figure 6).

### WWP1 depletion induced apoptosis in HCC cell lines

**Figure 7:**
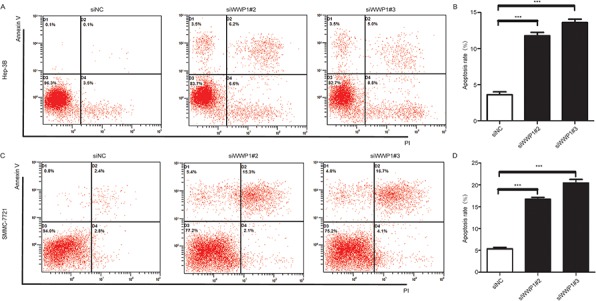
Effect of WWP1 on apoptosis in HCC cells Effect of WWP1 down-regulation on apoptosis in Hep-3B **A, B.** and SMMC-7721 **C, D.** cells 72 h after siRNA(s) transfection. Knockdown WWP1 significantly induced apoptosis. **P < 0.01 *versus* control; ***P < 0.001 *versus* siControl.

In order to examine how a deficiency in WWP1 might affect cell growth in HCC cell lines, flow-cytometric analysis with annexin V-FITC and PI was carried out in Hep-3B and SMMC-7721cells. The results showed that the percentages of apoptotic cells in the Hep-3B cells 72 h post-transfection with siWWP1#2 and siWWP1#3 increased by 9.5% and 10.3%, respectively, compared to those in negative (siNC) control cells (Figure 7A and 7B). Similar results were observed in SMMC-7721 cells, with percentage differences of 12.5% and 14.5%, respectively (Figure 7C and 7D).

## DISCUSSION

Ubiquitination is an inducible and reversible post-translational modification that plays key roles in many biological processes and physiological responses, including the regulation of cell cycle progression, DNA repair, signal transduction, protein stability, receptor transport, gene transcription and immune response [34]. It also tags proteins for degradation by plasma membrane proteins or 26S proteasome-mediated degradation, endocytosis and destruction in the lysosome [34]. Ubiquitination involves the formation of an isopeptide bond between the C-terminal Gly76 carboxyl group of ubiquitin and the ε-amino group of an internal Lys residue of a substrate. It is activated by a cascade of reactions catalyzed by three classes of enzymes: ubiquitin activating enzyme (E1), ubiquitin conjugating enzymes (E2) and ubiquitin-protein ligases (E3) [12]. Ubiquitin is first activated by E1 and ATP to form a high-energy thioester intermediate with E1; the activated ubiquitin is translated from E1 to the active site cysteine on one of several E2s; E2 then transfers the activated ubiquitin to a lysine residue on a target protein recognized by E3 [13]. E3s play pivotal roles in the ubiquitination pathway by directly catalyzing or facilitating the transfer of ubiquitin to their molecular targets, primarily to the Lys residues but in some cases to the N terminus [34]. E3s recognize specific substrates and modify protein substrates by monoubiquitylation or by sequential attachment of ubiquitin molecules to form polyubiquitin chains [13].

There are over 500 E3s in mammalian cells. Recent reports have shown that most single peptide E3s contain a novel RING finger domain or a HECT domain [15]. Genetic alterations, abnormal expression and dysfunctions in E3s have been implicated in the pathogeneses of a wide spectrum of human malignancies. WWP1 is a member of the E3 superfamily and its expression has been linked to prognosis in a variety human malignancies, suggesting that WWP1 may play a role as a tumor oncogene: WWP1 was significantly increased in oral cancer tissue specimens and cell lines [32]; increased levels of WWP1 mRNA and protein were reported in a subset of breast [30, 31] and prostate cancers [32]; and similar observation were made in head and neck squamous cell carcinomas [38]. Consistent with these studies, we found that WWP1 was upregulated in primary HCC tissues at both the transcriptional and translational levels. Although WWP1 has been shown to be upregulated in many human cancers, it has also been reported as downregulated in some other types of tumors [35, 36], suggesting that WWP1 plays distinct roles in different tumors. Therefore, details of its molecular mechanisms in cancer cells need to be elucidated.

Our study has provided the first evidence that increased expression of WWP1 may be linked to an unfavorable clinical outcome in patients with HCC. Our findings showed that WWP1 was abnormally elevated in primary HCC tissue specimens compared to normal hepatic tissues, and overexpression of WWP1 was significantly correlated with larger tumors, poorly differentiated histological grade, advanced TNM stage, vascular invasion, lower OS and PFS times, and poor prognosis in a cohort of 149 patients who had undergone surgery for primary HCC. In addition, Cox regression analysis indicated that WWP1 was an independent prognostic marker for OS in patients with HCC.

To further elucidate the role of WWP1 in the tumorigenesis of HCC, siRNAs were used to knockdown expression of WWP1 in two HCC cell lines, Hep-3B and SMMC-7721 cells. Colony formation and proliferation assays confirmed that silencing WWP1 inhibited cell growth; whereas migration and Matrigel invasion assays showed that it suppressed migration and invasion *in vitro*, indicating that WWP1 played a role in tumor metastasis. Further investigations by flow cytometry suggested that these effects may result in part to the induction of cell cycle arrest and apoptosis in HCC cells. A similar observation was reported by Li Zhang *et al*. who found that downregulation of WWP1 induced cell cycle arrest at G0/G1 phase and apoptosis in human gastric cancer cell lines [37]. Further support for the association between WWP1 and cell cycle was provided by Angelo Peschiaroli *et al*. who found that upregulation of WWP1 increased Δp63-dependent transcription, whereas depletion of WWP1 induced cell cycle arrest in human primary keratinocytes [38]; Nguyen Huu *et al*. found that expression of WWP1 inhibited apoptosis in breast and prostate cancer cells via the inhibition of TGF-β-mediated signaling [35]. In addition, several reports have suggested a potential role for WWP1 in various signaling pathways, including TGF-β [25, 26], p27Kip1 [39] and EGF [11, 28].

A major cause of poor prognosis in HCC is the lack of early diagnostic indicators; therefore, identifying novel and specific prognostic biomarkers for patients with early stage HCC is crucial [40]. At present, AFP is most commonly applied serological marker for surveillance, diagnosis and prediction of outcome in patients with HCC [41, 42, 43]. However, the diagnostic and prognostic sensitivity of AFP is poor in the early stages of the disease, especially when used isolation, and patients with low levels of AFP are often missed and progress to late stage HCC before becoming clinically symptomatic [43]. Over half of the patients (55.0%) in our study cohort exhibited AFP levels < 25 ng/ml; however, those patients with low stage HCC (TNM stage I) who also had high levels of WWP1 expression were found to have shorter survival times (OS, *P* < 0.001; DFS, *P* < 0.001) and an increased risk of recurrence. These results highlighted the potential value of WWP1 as a prognostic indicator in subgroups of patients with normal or low levels of AFP (≤ 25 ng/ml) or other indicators of early-stage HCC.

In summary, this study demonstrated that WWP1 possessed oncogenic properties when overexpressed; acted as a negative predictor of prognosis in patients with HCC; and that inhibiting WWP1 could suppress HCC cell growth, colony formation, migration and invasion, while inducing cell cycle arrest and apoptosis in HCC cells *in vitro*. These findings suggested that WWP1 might serve as a valuable prognostic biomarker and a novel therapeutic target in the treatment of patients with HCC.

## MATERIALS AND METHODS

### Cell lines and culture

HCC cell lines Hep3B (HB-8064), HepG2 (HB-8065) and SK-Hep1 (HTB-52) were purchased from American Type Culture Collection (ATCC; Rockville, MD, USA); Huh-7 (RCB 1366) was purchased from the RIKEN cell bank (Ibaraki, Japan); BEL-7402 was purchased from the Committee of Type Culture Collection of the Chinese Academy of Sciences (Shanghai, China). Human liver immortal cell line L02 was purchased from Biomics Biotechnologies (Nantong, China). All the cell lines were cultured in Dulbecco's modification of Eagle's medium (DMEM; Gibco; Grand Island, NY, USA) supplemented with 10% fetal bovine serum (FBS; Gibco) and 100 units/ml penicillin plus 100 μg/ml streptomycin. Cells were cultured at 37°C in a humidified atmosphere of 5% CO_2_.

### Patients and HCC tissue samples

A total of 149 paraffin-embedded tissue samples were randomly selected from patients who had undergone liver resection for HCC at the Sun Yat-sen University Cancer Center (SYSUCC) between November 2007 and December 2008. None of the patients had received preoperative treatment. Their median age was 47 years (range: 15–70 years) and the male:female ratio was 122:27. All diagnoses had been histologically confirmed independently by at least two experienced pathologists, primarily by examination of sections following H & E staining. Follow-up data, including clinical and laboratory examinations, were obtained for all patients every three months for the first two years, every six months for the following two years, and annually for the next five years or until death, whichever was sooner. International Union Against Cancer (UICC) TNM classification were used to verify the clinical stage[44]. Disease-free survival (DFS) was calculated from the date of surgery to the date of recurrence, metastasis, death or final follow-up; progression-free survival (PFS) was calculated from the date of surgery to progression, relapse, death or final follow-up. Overall survival (OS) was calculated from the date of surgery to the date of death or final follow-up.

In addition, 60 fresh tumor and adjacent non-tumor liver parenchyma were collected from patients who undergoing surgical resection for HCC at SYSUCC between 2012 and 2013. Histopathological diagnoses were confirmed by pathological examinations. Immediately following collection, the paired specimens were immersed in RNAlater solution (Ambion; Life Technologies; Carlsbad, CA, Inc., USA) to prevent RNA degradation, stored at 4°C overnight to allow RNAlater to fully penetrate into the tissues and then frozen at −80°C until required for experimentation.

### RNA extraction and real-time quantitative polymerase chain reaction (RT-qPCR)

Total RNA was extracted from the 60 freshly frozen paired tissue specimens using Trizol reagent (Invitrogen; Carlsbad, CA, USA) according to the manufacturer's protocol. RNA concentration and purity were determined by absorbance at 260 nm using a NanoDrop ND-1000 spectrophotometer (NanoDrop Technologies; Houston, TX, USA). Reverse transcription (RT) was performed on 2 μg of total RNA/sample using M-MLV reverse transcriptase (Promega; Madison, WI, USA) according to the manufacturer's instructions. Newly synthesized cDNA was amplified by RT-qPCR to enable the expression levels of WWP1 to be detected. The relative expressions of WWP1 were calculated by normalization against GAPDH. The primer sequences were as follows: WWP1, forward 5′-GGAGCTATGCAACAGTTTAACCAA-3′, reverse 5′-AAGTAAACCCTGTCTGTTGAATCCA-3′; GAPDH, forward 5′-GGAGATTGTTGCCATCAACG-3′, reverse 5′-TTGGTGGTGCAGGATGCATT-3′. RT-qPCR was performed using SYBR Green Master Mix with an ABI 7900HT real-time PCR system (Life Technologies). The thermal profile consisted of an initial denaturation step at 95°C for 10 min, followed by 40 cycles at 95°C for 30 s, and a final step at 60°C for 1 min. The melting curve was determined at 95°C for 15 s, 60°C for 15 s and 72°C for 15 s to confirm the specificities of the resulting products. The crossing threshold (Ct) value of each sample was calculated during the exponential amplification phase using the instrument's software (SDS v.2.3). Data were analyzed using the comparative threshold cycle (2-ΔΔCT) method. All experiments were performed in triplicate.

### Protein extraction and western blotting

Freshly frozen tissue specimens and cell lines were suspended in ice-cold RIPA lysis buffer (Beyotime Technology; Shanghai, China). Samples were centrifuged at 12,000 g for 30 min at 4°C and the supernatants were assayed to determine protein concentrations using a BCA Protein Assay Kit (Bio-Rad; Hercules, CA, USA). After quantification, 30 μg protein from each sample was denatured prior to 12% SDS-PAGE electrophoreses and then transferred to PVDF membranes (Bio-Rad). Non-specific binding was blocked by incubating the membranes in 8% nonfat milk for 1 h at room temperature. The membranes were then incubated overnight at 4°C with either rabbit anti-WWP1 (1:2000; Proteintech, Cat# 13587–1-AP) or rabbit anti-GAPDH (1:5000; Proteintech, Cat# 10494–1-AP) primary antibodies. After washing in phosphate-buffered saline-Tween (PBST), the membranes were incubated with Horseradish Peroxidase (HRP)-conjugated goat anti-rabbit antibody (1:5000; Calbiochem, Cat# AP307P) at room temperature for 1 h, then washed against in PBST. The immunoreactive proteins were visualized using enhanced chemiluminescence (ECL) detection reagent with an ECL kit (Cell Signaling Technology, Cat# 7003) and. The optical densities of the protein bands were measured using Quantity One software (Bio-Rad).

### Immunohistochemistry staining

The 149 paraffin-embedded HCC tissue specimens were cut into 2 μm sections and heated at 60°C for 2 h. The sections were deparaffinized in Xylene and rehydrated through descending alcohol concentrations. They were washed in PBS (pH 7.4) before being processed in EDTA (1 mM, pH 8.0) in a microwave oven at 100°C for 15 min to expose antigenic sites, then cooled for 1 h at room temperature. Endogenous peroxidase activity was blocked using 0.3% hydrogen peroxide for 15 min at room temperature. The sections were incubated at 4°C overnight in a humidified chamber with primary antibody rabbit anti-WWP1 (1:800, Proteintech, Cat# 13587–1-AP). After washing in PBS, they were incubated with HRP-conjugated secondary antibody using an Envision Detection Kit, GK500705 (Gene Tech; Shanghai, China) for 30 min at room temperature. A negative control was prepared by replacing the primary antibody with PBS. Finally, the sections were incubated with 3,3′-diaminobenzidine (DAB) and counterstained with hematoxylin and eosin (H&E) before being examined by light microscopy.

The resulting slides were independently assessed by two of the authors and scored according to their staining intensity and percentage of positive staining as follows: the intensity of staining was classified as 0 (no staining), 1 (weak staining), 2 (moderate staining) and 3 (strong staining); the percentage of positive staining was defined as 0 (0–5%, negative), 1 (5%–25%, sporadic), 2 (25%–50%, focal), or 3 (≥ 50%, diffuse). The two values were multiplied to give final scores between 0 and 6. These were used to define the expressions of WWP1 as follows: “−” (negative, score 0–1), “+” (weakly positive, score 2–3), “++” (positive, score 4–5), “+++” (strongly positive, score 6). Samples classed as “−” or “+” were defined as having low WWP1 expression, while those classed as “++” and “+++” were defined as having high WWP1 expression.

### Transfection and short-interfering RNAs (siRNA)

Knockdown of WWP1 in the HCC and normal hepatic cell lines was achieved using two different types of siRNAs that specifically targeted WWP1 (siWWP1). A negative control siRNA (siNC) was also prepared. The siRNAs were synthesized by GenePharma (Shanghai, China). Transfection was carried out with 600 pmol siWWP1 or siNC using Lipofectamine RNAi MAX reagent (Invitrogen; Carlsbad, CA, USA) according to the manufacturer's protocol. Briefly, 2 × 10^6^ cells from Hep-3B and SMM-7721 cell lines were transfected under serum-free and antibiotic-free conditions for 8 h. The medium was changed and the cells were either incubated for 48 h prior to colony formation and cell proliferation analysis; or for 72 h prior to cell cycle and apoptosis analyses. The effective siRNA sequences (sense and antisense) were as follows: siWWP1#2, 5′-CCUAUUAUGUGGAUCAUAATT-3′, and 5′-UUAUGAUCCACAAUAAUAGGTT-3′; siWWP1#3, 5′-GUGGAAGGUUGCAGUUACATT-3′, and 5′-UGUAACUGCAACCUUCCACTT-3′ and siNC, 5′-UUCUCCGAACGUGUCACGUTT-3′ and 5′-ACGUGACACGUUCGGAGAATT-3′.

### Colony formation assay

Colony formation assays were carried out to assess the viability of HCC cells following transfection with siWWP1 or siNC for 48 h. The transfected cells were plated in 6-well plates at a density of 800 cells/2 ml well and incubated at 37°C in a humidified atmosphere of 5% CO_2_ for 14 days. The cells were washed with PBS and fixed in 75% ethanol at room temperature for 10 min before being stained with 0.5% crystal violet for a further 10 min. The numbers of stained cells were counted and those colonies that contained > 50 cells were classed as clones. Colony-forming efficiency was expressed as a ratio of the number of colonies formed to the number of cells seeded.

### Cell proliferation assay

Cell proliferation assays were performed to evaluate the proliferation capabilities of HCC cells following transfection with siWWP1 or siNC for 48 h. In brief, the transfected cells were seeded into 96-well plates at a density of 1 × 10^3^ cells/well and their growth rates were evaluated using an MTS cell proliferation kit (Promega; Madison, WI, USA) with methanethiosulfonate (MTS) reagent, according to the manufacturer's instructions. Each assay was performed in triplicate.

### Cell cycle analysis by propidium iodide (PI) flow cytometry

Cell cycle analysis was carried out by flow cytometry using propidium iodide (PI). In brief, HCC cells were collected following transfection with siWWP1 or siNC for 72 h. The cells were washed by centrifugation in ice-cold PBS for 5 min at 125 × g, then fixed in 75% ethanol at −20°C overnight, then treated with RNase at 37°C for 30 min prior to being stained with PI (Bestbio, Shanghai, China) for 60 min in the dark at 4°C. Cell cycle distribution was determined using a flow cytometer (Beckman Coulter; Fullerton, CA, USA) according to the manufacturer's instructions.

### Apoptosis assay by annexin V-FITC and PI flow cytometry

Apoptosis assays were performed by flow cytometry using annexin V-FITC and PI. In brief, HCC cells were harvested after being transfected with siWWP1 or siNC for 72 h. They were washed twice with ice-cold PBS and then stained with Annexin V-FITC and PI according to the manufacturer's instructions. Analysis was performed a flow cytometer (Beckman Coulter) according to the manufacturer's instructions.

### Cell migration and invasion assays

Cell migration and invasion assays were carried out using 24-well transwell chambers with an 8 μm pore polycarbonate membrane insert (Corning Incorporated, Corning, NY, USA). For the migration assays, the cells were seeded into the upper chambers (without Matrigel) at a density of 1 × 10^5^; for the invasion assays, the cells were seeded at a density of 2 × 10^5^ on the top side of the membrane pre-coated with Matrigel (BD, Franklin Lakes, NJ, USA). For both assays, 200 μL RPMI 1640 medium supplemented with 0.5% FBS was added to the upper chambers and 600 μL 10% FBS-1640 was added to the lower wells. The assays were carried out for 24 h (migration) or 48 h (invasion) at 37°C. Cells that migrated or invaded to the bottom of the upper membrane were collected mechanically, fixed in 75% ethanol and stained with 0.05% crystal violet in methanol. Migration or invasion efficiency was determined by microscopy: ten fields were randomly selected from three wells (30 fields in total) for each assay. Each experiment was performed in triplicate.

### Statistical analysis

Statistical analyses were carried out using SPSS v16.0 statistical software (SPSS, Chicago, IL, USA). A paired-sample *t*-test was used to evaluate differences in WWP1 mRNA or protein expression levels between the HCC specimens and their matched adjacent non-tumor tissues. Pearson's chi-square test (χ^2^) was used to determine the correlations between WWP1 expression levels and the clinical characteristics of patients with HCC. The Kaplan-Meier method and log-rank test were used to evaluate survival curves for OS, DFS and PFS. The Cox proportional-hazard analysis was used for univariate and multivariate analyses to explore the effect of the clinicopathological variables and WWP1 expression on survival. Only the factors which were found to have statistically significant associations with overall survival based on a univariate analysis would be included in a multivariate Cox proportional hazards model to adjust for the effects of the covariates. Furthermore, variables that were highly associated with others were excluded from the final multivariate Cox proportional hazards model. A two-tailed unpaired Student *t*-test was used to assess differences in cell proliferation rates, colony formation, cell cycle distribution, apoptotic frequency and cell migration and invasion between siWWP1- and siNC-transfected HCC cells. Statistical differences from at least three independent experiments were expressed as mean ± standard deviation (SD). A two-sided *P*-value < 0.05 was considered to be statistically significant.
